# The changing face of maxillofacial trauma during the 2020 COVID-19 lockdowns in Melbourne, Australia

**DOI:** 10.1007/s10006-022-01041-6

**Published:** 2022-01-23

**Authors:** Sipho Simon Nhongo, Anton Sklavos, Kai Lee, Steven T. F. Chan, Stephen Austin

**Affiliations:** 1 Division of Maxillofacial Surgery, Footscray Hospital, 160 Gordon St, Footscray, VIC 3011 Australia; 2 Division of Maxillofacial Surgery, University Hospital Geelong, Bellerine St, Geelong, VIC 3220 Australia; 3 Consultant Oral & Maxillofacial Surgeon, Dentistry & Oral Health, LaTrobe University, Plenty Rd &, Kingsbury Dr, Bundoora, VIC 3086 Australia; 4 Professor of Surgery, Western Clinical School, The University of Melbourne, 176 Furlong Road, St Albans, , Victoria 3021 Australia; 5 Division of Maxillofacial Surgery, Footscray Hospital, 160 Gordon St, Footscray, Melbourne, VIC 3011 Australia

**Keywords:** Maxillofacial injuries, Jaw fractures, Violence, COVID-19, Coronavirus, Public health

## Abstract

**Purpose:**

To compare the incidence, aetiology, and patterns of maxillofacial fracture presentations during the various stages of the 2020 Melbourne COVID-19 lockdown restrictions to periods outside lockdown in 2019 and 2020.

**Methods:**

This is a retrospective study of 344 subjects. The patterns of facial trauma presentations to a tertiary hospital in metropolitan Melbourne during the 2020 COVID-19 restrictions were compared to periods with no restrictions over 22 months from March 2019 to December 2020.

**Results:**

The incidence of maxillofacial fractures decreased by 28% during lockdown (0.41 vs. 0.57 injuries/day, *P* = 0.0003). Falls overtook interpersonal violence as the leading cause of fractures (44% of lockdown presentations vs. 25.7% of presentations outside lockdown, *P* = 0.002), while sporting injuries dropped drastically (4% vs. 17.1%, *P* = 0.005). Lockdowns saw an increase in the proportion of female patients (40% vs. 26.8%, *P* = 0.03) and a fivefold increase in proportion of domestic violence-related fractures (6.7% vs. 1.1%, *P* = 0.006). Alcohol-related injuries decreased significantly (11% vs. 21%, *P* = 0.03).

**Conclusions:**

While restrictions reduced rates of interpersonal violence and alcohol-related maxillofacial trauma, there was a higher proportion of injuries to females, increased falls, and domestic violence-related injuries.

## Introduction

Maxillofacial trauma is a consequence of human behaviour. Epidemiological studies have shown that maxillofacial fractures are most commonly a consequence of interpersonal violence, road traffic accidents, falls, sports-related injuries, and work-related injuries [1–4]. Typically, patients are middle-aged males [2, 3]. Alcohol consumption has long been identified as a major risk factor for maxillofacial trauma, increasing the risk of interpersonal violence and motor vehicle accidents resulting in fractures [5, 6]. Alcohol also increases the severity of the injuries and results in more injuries requiring operative management [5, 7].

The COVID-19 pandemic along with the mass public health regulations and social restrictions fundamentally changed patterns of human activity and behaviour at a population level. On the 16th of March 2020, Australia declared a public health emergency in response to the growing number of community-acquired cases of SARS-CoV-2 [8]. The state of Victoria—particularly metropolitan Melbourne—was hit the hardest, recording the most cases, deaths, and the highest transmission rate in Australia [9, 10]. The first lockdown was introduced on the 22nd of March until the 31st of May 2020. Restrictions eased between the 31st of May and the 8th of July, before a second lockdown period extending until the 26th of October. A large proportion of the metropolitan workforce began working from home, vastly decreasing the use of public and private transportation. Restrictions included the shutting down of licenced venues (bars, clubs, restaurants, cafés) and the prohibition of public gatherings and sporting events—precluding the consumption of alcohol in such settings.

The purpose of this study is to identify and examine changes in the patterns of facial trauma presentations to a tertiary hospital in metropolitan Melbourne during the 2020 COVID-19 restrictions, compared to periods with no restrictions over 22 months from March 2019 to December 2020. Of particular interest are changes in the incidence of injury, mechanism of injury, alcohol involvement, and numbers requiring operative management.

## Materials and methods

Ethical approval of the study was obtained from the Western Health human research ethics panel (Project number QA2020.89, ERM ID number 68382). The requirement for informed consent was waived by the ethical board owing to the retrospective study design and de-identified patient data. Privacy and confidentiality of all clinical information were maintained as per the World Medical Association Declaration of Helsinki.

### Study design, setting, and participants

This retrospective comparative study analysed the data of patients referred to the Western Health Maxillofacial Surgery unit for assessment and management of traumatic injuries between 23 March 2019 and 31 December 2020. Existing electronic medical records (including relevant imaging) of inpatient and outpatient presentations were reviewed. Inclusion criteria were (1) patients who presented between 22 March 2019 and 31 December 2020 and (2) patients who presented with traumatic injuries coded as S02.0 through S02.9 as defined by the International Classification of Disease, 10th Edition (ICD-10).

Patients presenting during the two Melbourne lockdowns (22nd of March–31st of May 2020, and 31st of May–8th of July 2020) were compared to those presenting outside of lockdown restrictions.

### Variables

The primary predictor variable was the timing of injury: during lockdown (22 March 2020–31 May 2020, 8 July–27 October 2020) compared to the outside lockdown between 22 March 2019 and 31 December 2020. The primary outcome variable was the incidence of maxillofacial fractures. Demographic study variables included age at the time of injury and gender. Other variables included fracture diagnosis and site, number of fractures per patient, mechanism of injury, alcohol-related injury, other illicit substance use, and treatment modality (operative vs. nonoperative).

### Data collection

Data were collected manually from the electronic medical records. The following data was de-identified:

1. Demographics: age, gender
2. Injury history: date of injury, mechanism of injury, alcohol intoxication at the time of injury, intoxication with other illicit substances at the time of injury, number of fractures per injury, and anatomical site(s) of injury
3. Treatment: operative vs. nonoperative

### Statistical analysis

Descriptive data were presented as the median and interquartile range (IQR), and continuous data were analysed with Mann–Whitney *U* statistics. Contingency table analyses of differences in binomial proportions were performed by permutation exact methods using StatXact v9 Cytel Software Corporation, USA. All statistical tests were two-sided, and the 5% *α*-level was used to assess significance.

## Results

A total of 344 patients (242 male and 102 female) presented with traumatic maxillofacial fractures between 22 March 2019 and 31 December 2020.

### Trauma incidence

**Fig. 1:**
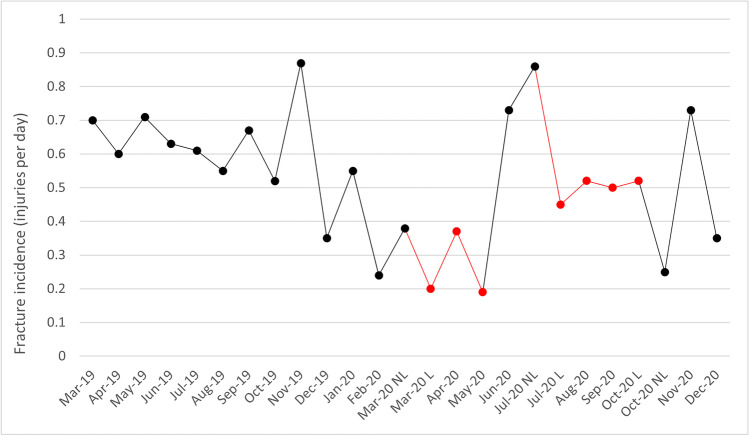
Monthly incidence of maxillofacial fractures. Lockdown (L) in red vs. no lockdown (NL) in black

Figure 1 illustrates the timeline of monthly fracture rates (injuries per day). During lockdowns (22 March 2020–31 May 2020, 8 July–27 October 2020), there were 75 maxillofacial fractures over 181 days (0.41 injuries per day), while there were 269 fractures over 470 days outside lockdown (0.57 injuries per day). This decrease in the incidence of injuries was shown to be significant through an exact test of binomial proportions (*P* = 0.0003).

### Demographics and injury characteristics

Patient demographics and injury characteristics are compared between the lockdown (L) and non-lockdown (NL) groups in Table 1. The majority of subjects in both the L and NL groups were male; however, the proportion of male subjects during lockdown decreased significantly (60% in lockdown compared to 73.2% with no restrictions, *P* = 0.03).

**Table 1:** Comparison of demographics, mechanism of injury, and fracture type

	Lockdown: 181 days (*n* = 75)	Non-lockdown: 470 days (*n* = 269)	P value
Demographics			
Gender^†^			0.03
Male	45 (60.0%)	197 (73.2%)	
Female	30 (40.0%)	72 (26.8%)	
Age^§^	40 (IQR 27–63)	35 (IQR 25–58)	0.28
Mechanism of injury			
Interpersonal violence^†^	24 (32%)	117 (43.5%)	0.07
Falls^†^	33 (44%)	69 (25.7%)	0.002
Mechanical	18 (24%)	42 (15.6%)	0.09
Intrinsic	15 (20%)	27 (10%)	0.02
Intoxicated fall^†^	2 (3%)	9 (3.3%)	0.93
Domestic violence^†^	5 (6.7%)	3 (1.1%)	0.006
Sporting injury^†^	3 (4%)	46 (17.1%)	0.005
Motor vehicle accident^†^	2 (2.7%)	3 (1.1%)	0.96
Motorbike accident	0	2 (0.7%)	
Workplace injury	0	3 (1.1%)	
Other transport injury^†^	1 (1.3%)	12 (4.5%)	
Seizure^†^	1 (1.3%)	1 (0.4%)	0.44
Accident (other) ^†^	1 (1.3%)	4 (1.4%)	0.90
Unclear	3 (4%)	2 (0.8%)	
Fracture type			
Orbit	22 (29.3%)	76 (28.3%)	0.86
Nasal bones	19 (25.3%)	48 (17.8%)	0.15
Mandible	16 (21.3%)	53 (19.7%)	0.76
ZMC	12 (16.0%)	61 (22.7%)	0.23
Maxilla	10 (7.5%)	28 (10.4%)	0.48
Zygomatic arch	4 (5.3%)	12 (4.5%)	0.82
Frontal bone	3 (4.0%)	6 (2.2%)	0.48
NOE	0	7 (2.6%)	
Le Fort I	0	2 (0.7%)	
Le Fort II	0	2 (0.7%)	

^†^Differences in binomial proportions; ^‡^median (*IQR*, interquartile range); ^§^Mann–Whitney *U* test; pushbike accidents and scooters

Abbreviations: *ZMC*, zygomaticomaxillary complex; *NOE*, nasoorbitoethmoid

Interpersonal violence and falls were consistently the 2 leading causes of fractures in both L and NL cohorts. There was however a significant increase in the proportion of maxillofacial fractures caused by falls (44% from 125.7%, *P* = 0.002).

Domestic violence as a percentage of presentations rose significantly during the lockdown, accounting for 6.7% of injuries, compared to only 1% outside lockdown, *P* = 0.006. Sporting injuries were reduced by more than 93% during lockdown (*P* = 0.005).

### Alcohol-related injuries

Table 2 compares alcohol-related injuries in lockdown versus periods with no restrictions. The proportion of alcohol-related injuries saw a significant decline—from 21 of all presentations out of lockdown to just 11% of presentations during lockdown (*P* = 0.03).

**Table 2:** Comparison of alcohol and substance-related injuries

	Lockdown: 181 days (*n* = 75)	Non-lockdown: 470 days (*n* = 269)	P value
Alcohol			
Alcohol-related injury^†^	8 (11%)	57 (21%)	0.03
Non-alcohol related injury^†^	57 (76%)	171 (64%)	
Unclear^†^	10 (13%)	41 (15%)	0.71
Other illicit substances*			
Intoxicated^†^	10 (13.3%)	14 (5.2%)	0.02
Not intoxicated^†^	65 (86.6%)	255 (94.8%)	

^†^Differences in binomial proportions

With both L and NL cohorts combined, alcohol-related injuries significantly increase the likelihood of operative management: 40% of alcohol-related injuries required surgical management compared to 24% of non-alcohol-related injuries (*P* = 0.03).

### Management outcomes

Management outcomes are shown in Table 3. During the lockdown 24% (18/74) of traumatic fractures required operative management, similar to the 27.5% (74/269) rate of operative management outside lockdown. Emergency cases accounted for 61% of operations during the lockdown, compared to 48.6% outside lockdown; however, this was not found to be statistically significant (*P* = 0.51). Review attendance did not change appreciably, with an 89.3% attendance rate during the lockdown and a 91.8% attendance rate outside lockdown (*P* = 0.53).

**Table 3:** Management outcomes

	Lockdown (n = 75)	No lockdown (n = 269)	P value
Review outcome			
Attended review^*†*^	67 (89.3%)	247 (91.8%)	0.51
Did not attend^*†*^	8 (10.7%)	22 (8.2%)	
Operative cases			
Operative^*†*^	18 (26.9%)	74 (30.0%)	0.65
Emergency	11 (61.1%)	36 (48.6%)	0.53
Elective	7 (38.9%)	38 (51.4%)	
Nonoperative^*†*^	49 (73.1%)	173 (70.0%)	

^†^Differences in binomial proportions

## Discussion

This study sought to determine if there was a difference in the patterns of maxillofacial fracture presentations to a metropolitan trauma service in Melbourne, Australia, during the COVID-19 lockdowns of 2020. Internationally thus far, evidence exploring the epidemiological effect of coronavirus restrictions on maxillofacial injuries is sparse—the authors identified 2 North American studies, 1 French, and a combined Australian–UK study [8, 11–13]. The consistent finding worldwide has been a decrease in the incidence of maxillofacial injuries during lockdowns. This study confirms this, with a 28% decrease in the incidence of maxillofacial fractures during lockdown (*P* = 0.0003).

There was also a notable change in the causality of injury: the proportion of injuries resulting from falls increased during lockdowns, while the proportion of injuries resulting from interpersonal violence and sporting injuries declined significantly. This, too, was consistent with existing evidence [8, 12, 13].

A demographic paradigm shift took place in lockdown, with this study finding the proportion of female patients significantly increased during the lockdown period, constituting 40% of patients with maxillofacial fractures during lockdown compared to 26.8% outside lockdown. Furthermore, female subjects accounted for 100% of the domestic violence-related (DV) presentations during lockdown which, sadly, increased fivefold in proportion compared to outside lockdown (6.7% compared to 1.1% outside lockdown, *P* = 0.006). This finding is corroborated by the Victorian Crime Statistics Agency report of a 9% increase in family violence incidents brought to the police in 2020 compared to 2019 [14]. Even more alarming is that DV-related injuries may be underestimated due to increased barriers to help-seeking given stay-at-home orders and social distancing.

The sequelae of social restrictions, home confinement, and isolation include increased incidence of mental health-related issues, difficulty in finding appropriate medical care and medications, and psychological stress associated with financial loss and social isolation [15]. These stressors are likely contributors to the observed increase in domestic violence. People may also be more prone to self-medication, as suggested by the doubling in the proportion of patients intoxicated with other substances during lockdown (13.3% compared to 5.2% outside lockdown, *P* = 0.02).

One fortuitous by-product of restrictions was a significant decrease in the incidence and overall proportion of alcohol-related injuries, with the rate of alcohol related maxillofacial fractures outside of lockdown being three times greater than during the lockdown periods. In Australia, the overall purchasing of alcohol during the COVID-19 lockdowns did not significantly change [16]; however, the closure of licenced premises reduced consumption of harmful levels of alcohol [17]. The pattern of alcohol-related injuries in this study suggests that alcohol consumption alone may not be sufficient to cause increases in maxillofacial trauma, and that opening of licenced premises and gathering of crowds are essential components in the aetiology of interpersonal violence.

An Australian–UK study suggested that rates of interpersonal violence decreasing during the COVID-19 lockdown period may have been a consequence of the closure of licenced premises resulting in reduced sale and consumption of alcohol; however, they did not present data on alcohol-related maxillofacial fractures [8]. A Seattle-based study by Ludwig et al. produced conflicting results, as they reported an increase in the proportion of interpersonal violence-related injury [10]. However, this study was in a population in Seattle Washington during a time of civil unrest and rioting, and this may have impacted their numbers of interpersonal violence.

One limitation of this study is sample size—over the 181-day lockdown period, there were only 75 presentations of maxillofacial fractures. However, this sample size is larger than previous studies: one over a 7-week period with only 38 patients [13] and another over an 8-week period of 73 cases in an Australian population and 37 patients in a UK population [8]. Larger studies include a multicentre French study of 106 presentations [11] and a study from Washington, USA, of 235 maxillofacial fractures [12].

Lastly, patient and injury characteristics were obtained retrospectively from medical records, thus reliant on clinician notes. Prospective, standardised data collection would improve the accuracy of results and reliable comparability.

The findings from this study show that the COVID-19 restrictions implemented in Melbourne, Australia, in 2020 had a significant impact on the patterns of maxillofacial trauma presenting to our institution. While restrictions on licenced premises reduced rates of interpersonal violence and alcohol-related maxillofacial trauma, this appeared to come at the cost of a higher proportion of injuries to females, increased falls, and domestic violence-related cases. Also noted was an increased proportion of injuries sustained under the influence of illicit substances. The unintended consequences of social restrictions made necessary by the 2020 COVID-19 pandemic serve as a harbinger for just some of the future challenges we as clinicians and as a society are likely to face moving forward.

## Data Availability

The datasets used and analysed during the current study are available from the corresponding author on reasonable request.
